# Nitroglycerine triggers triptan-responsive cranial allodynia and trigeminal neuronal hypersensitivity

**DOI:** 10.1093/brain/awy313

**Published:** 2018-12-27

**Authors:** Simon Akerman, Nazia Karsan, Pyari Bose, Jan R Hoffmann, Philip R Holland, Marcela Romero-Reyes, Peter J Goadsby

**Affiliations:** 1 Headache Group-Department of Neurology, University of California, San Francisco, San Francisco, CA, USA; 2 Department of Oral and Maxillofacial Pathology, Radiology and Medicine, New York University College of Dentistry, New York, NY, USA; 3 Department of Neural and Pain Sciences, University of Maryland Baltimore, Baltimore, Maryland, MD, USA; 4 Headache Group, Department of Basic and Clinical Neuroscience, Institute of Psychology, Psychiatry and Neuroscience, King’s College London, London, UK; 5 NIHR-Wellcome Trust King’s Clinical Research Facility, King’s College Hospital, London, UK

**Keywords:** nitroglycerine, cutaneous allodynia, trigeminovascular, hypersensitivity, central sensitization

## Abstract

Cranial allodynia associated with spontaneous migraine is reported as either responsive to triptan treatment or to be predictive of lack of triptan efficacy. These conflicting results suggest that a single mechanism mediating the underlying neurophysiology of migraine symptoms is unlikely. The lack of a translational approach to study cranial allodynia reported in migraine patients is a limitation in dissecting potential mechanisms. Our objective was to study triptan-responsive cranial allodynia in migraine patients, and to develop an approach to studying its neural basis in the laboratory. Using nitroglycerine to trigger migraine attacks, we investigated whether cranial allodynia could be triggered experimentally, observing its response to treatment. Preclinically, we examined the cephalic response properties of central trigeminocervical neurons using extracellular recording techniques, determining changes to ongoing firing and somatosensory cranial-evoked sensitivity, in response to nitroglycerine followed by triptan treatment. Cranial allodynia was triggered alongside migraine-like headache in nearly half of subjects. Those who reported cranial allodynia accompanying their spontaneous migraine attacks were significantly more likely to have symptoms triggered than those that did not. Patients responded to treatment with aspirin or sumatriptan. Preclinically, nitroglycerine caused an increase in ongoing firing and hypersensitivity to intracranial-dural and extracranial-cutaneous (noxious and innocuous) somatosensory stimulation, reflecting signatures of central sensitization potentially mediating throbbing headache and cranial allodynia. These responses were aborted by a triptan. These data suggest that nitroglycerine can be used as an effective and reliable method to trigger cranial allodynia in subjects during evoked migraine, and the symptom is responsive to abortive triptan treatments. Preclinically, nitroglycerine activates the underlying neural mechanism of cephalic migraine symptoms, central sensitization, also predicting the clinical outcome to triptans. This supports a biological rationale that several mechanisms can mediate the underlying neurophysiology of migraine symptoms, with nitrergic-induced changes reflecting one that is relevant to spontaneous migraine in many migraineurs, whose symptoms of cranial allodynia are responsive to triptan treatment. This approach translates directly to responses in animals and is therefore a relevant platform to study migraine pathophysiology, and for use in migraine drug discovery.

## Teaser

There are limited experimental approaches to study cranial allodynia in migraine. Akerman et al. report that nitroglycerine triggers cranial allodynia alongside migraine-like headache in patients, and dural-intracranial trigeminal neuronal hypersensitivity in rats, with both responding to abortive treatments. Using nitroglycerine thus represents a reliable translational approach to study allodynia in migraine.

**See De Icco and Tassorelli (doi:10.1093/brain/awy315) for a scientific commentary on this article.** 

## Introduction

Migraine headache is thought to be the result of abnormal activation and sensitization (Noseda and Burstein, 2013), or the perception of activation (Akerman *et al.*, 2011; Goadsby *et al.*, 2017), of trigeminal primary afferents innervating the nociceptive producing dural vasculature and their central projections to the medullary dorsal horn and upper cervical spinal cord (Noseda and Burstein, 2013; Goadsby *et al.*, 2017). Sensitization of these dural-responsive trigeminovascular neurons, which also receive inputs from facial cutaneous areas, is thought to cause extracranial facial hypersensitivity manifesting as cutaneous allodynia and hyperalgesia in migraine patients (Burstein *et al.*, 2000). These symptoms affect up to two-thirds of migraine patients (Selby and Lance, 1960; Lipton *et al.*, 2008). Studies in migraineurs offer unique insights into the importance of trigeminovascular sensitization in migraine pathophysiology, with the presence and severity of cranial allodynia being a marker of susceptibility to chronic migraine (Bigal *et al.*, 2008).

As a symptom, cranial allodynia is an important substrate against which to evaluate abortive treatment response, both clinically and preclinically. This is because patient responses to acute treatments vary. In some reports patients with allodynia remain responsive to triptans (Cady *et al.*, 2007; Schoenen *et al.*, 2008; Diaz-Insa *et al.*, 2011), whereas in others the presence of cranial allodynia predicts the lack of triptan efficacy (Burstein *et al.*, 2004; Jakubowski *et al.*, 2005*a*). These conflicting outcomes suggest that the underlying mechanism of cranial allodynia in migraine cannot be fully explained by a single process. The lack of an effective and reliable translational approach to study cranial allodynia is a limitation in dissecting the mechanisms involved, with current studies relying on capturing spontaneous attacks while patients are visiting the headache clinic. Existing human migraine models, using exogenously administered substances, provide a unique and attractive opportunity to study the disorder (Ashina *et al.*, 2017). Nitroglycerine is the most established method to study migraine, triggering a delayed migraine-like headache in a high proportion of migraineurs (Iversen *et al.*, 1989), alongside non-painful premonitory symptoms (Afridi *et al.*, 2004; Maniyar *et al.*, 2014). It also produces activation in brain areas on functional brain imaging, similar to those demonstrated during spontaneous migraine (Bahra *et al.*, 2001; Afridi *et al.*, 2005). However, other phenotypic symptoms, such as cranial allodynia, have not been captured previously in such studies.

Here our aim was to determine whether nitroglycerine can trigger cranial allodynia associated with migraine, and subsequently the effectiveness of standard migraine abortive treatment with aspirin or sumatriptan, to relieve these symptoms. We also wanted to determine the translational potential of nitroglycerine as a trigger of migraine-associated cranial allodynia, by studying its neural basis and efficacy as a preclinical model. Nitroglycerine in rodents induces neuronal activation in similar brain areas (Tassorelli and Joseph, 1995; Koulchitsky *et al.*, 2004), and produces hind-paw and cranial cutaneous allodynia (Pradhan *et al.*, 2014; Marone *et al.*, 2018). However, its ability to trigger neurophysiological mechanisms within central trigeminovascular neurons that manifest as the neural mechanism of cephalic symptoms is unknown. Using electrophysiological recording of central trigeminocervical neurons, we analysed the neurophysiological changes caused by nitroglycerine on spontaneous firing and whether this manifested as neuronal hypersensitivity to somatosensory cranial stimulation. To validate this approach, we used delayed abortive treatment with a triptan to determine if it also predicted the clinical outcomes observed in both spontaneous migraine, and the nitroglycerine human model.

## Materials and methods

### Clinical studies

#### Study population and screening process

The study was carried out with research ethics approval by Camden and King’s Cross Ethics Committee (reference 15/LO/2241), and had Research and Development Approval at King’s College Hospital. Telephone interviews were conducted to prescreen patients (*n* = 335) that responded via e-mail to online and paper bulletins within migraine-related media. Inclusion and exclusion criteria (Supplementary Table 1), including age (18–50 years), diagnosis for migraine with or without aura (Headache Classification Committee of the International Headache Society, 2013), elimination of possible secondary cause of headache, presence of another chronic pain disorder or confounding medical condition, previous response to abortive medications used in the study, and contraindications to nitroglycerine infusion, were reviewed. Eligible patients (*n* = 53) were then invited to a first visit (Visit 1), where a study investigator conducted a full medical and family history, headache features and associated symptoms, with brief physical examination. Allodynia history was asked as a single question ‘Do you have abnormally high sensitivity of the skin of your scalp to touch during a migraine attack?’, and the response explored by a trained headache physician for clarity. Subjects meeting all criteria were explained they would be exposed to nitroglycerine infusion that might trigger migraine symptoms, and they were given an opportunity to ask any further questions, prior to providing informed consent.

#### Study design and procedures

Overall subjects made three or four study visits, depending on treatment arm: sumatriptan or aspirin, respectively, including placebo visit (vehicle for nitroglycerine), and the remainder involved migraine triggering with nitroglycerine. The reason for the mixed visit design was based on the initial study testing triptan response only. Another aspirin/placebo arm was included because of triptan non-responders based on spontaneous attacks. On Visit 1 subjects were entered into either treatment group, based on their preferred and usual successful response to each agent or drug class: triptan or non-steroidal anti-inflammatory drug (NSAID), with spontaneous attacks, and relative contraindications. Sumatriptan was chosen as it is the most commonly prescribed of the triptans and is available in injectable form with rapid onset of action, and aspirin similarly as it can also be administered intravenously, and because it is a highly effective NSAID for migraine abortion (Weatherall *et al.*, 2010). Subjects who had previously not responded to triptans or NSAID medications were excluded. Nitroglycerine (0.5 µg/kg/min) was intravenously infused over 20 min via the hand, forearm or antecubital fossa, using a 20–22G standard intravenous cannula. Subjects remained flat during infusion to limit nitroglycerine-induced hypotension, and were asked not to eat and drink in case of nausea and vomiting accompanying the migraine attack. Hydration was provided through a 125 ml/h, 5% dextrose/0.9% normal saline infusion following nitroglycerine. Subjects were monitored for the development of migraine headache and associated symptoms, along with blood pressure and heart rate prior to infusion, every 5 min during infusion, and subsequently every 15 min following infusion. If/when migraine-like headache reached moderate-severe intensity, subjects were treated with either sumatriptan [6 mg, subcutaneously (s.c.)] or aspirin [1 g, intravenous (i.v.)]. Subjects with successful nitroglycerine-triggered migraine-like headaches were invited back for subsequent visits, where they were exposed again to nitroglycerine or placebo. All conditions and questioning/monitoring were the same on each visit. This timeline is summarized in Supplementary Fig. 1. For the purposes of treating migraine, as the initial study was designed for a functional neuroimaging study, there was a delay in treating headache when it did reach moderate-severe intensity. In addition, consistent with other nitroglycerine provocation studies in the literature, the migraine headache ensued at a delayed time point following nitroglycerine infusion (Ashina *et al.*, 2017).

On all subsequent visits following Visit 1, all subjects were either exposed to nitroglycerine or placebo as the ‘triggering’ agent in a double-blind randomized design. This design was used to ensure that the symptoms experienced by subjects following infusion were truly attributable to the nitroglycerine-induced migraine process rather than a placebo effect.

#### Patient questioning and monitoring

Each subject was monitored in the facility for at least 5 h after the start of the infusion for the development of headache symptoms, and their subsequent response to abortive medication, until complete resolution of pain. To allow direct comparison of migraine phenotype, a standardized physician-administered symptom questionnaire of headache and associated features, including those of premonitory, aura, allodynia, vertigo, and postdrome, was used to collect data regarding previous spontaneous attacks, and the evolution of triggered attacks, across each study visit. In particular, as migraine-like headache reached moderate-severe intensity, subjects were asked: (i) ‘Do you have abnormally high sensitivity of the skin of your scalp to touch at the moment?’; and (ii) ‘Does this feel like the sensation you experience during one of your spontaneous attacks?’

Subjects were monitored until complete pain resolution. An improvement in allodynia was documented in answer to the question: ‘Has the sensitivity of the skin on your scalp completely resolved?’ An answer ‘yes’ was documented as treatment response, and any residual allodynia after treatment was documented as a failure of treatment.

Migraine headache was defined as suggested by the modified criteria for a pharmacologically provoked experimental migraine headache (Ashina *et al.*, 2013), summarized in Table 1.

**Table 1:** The modified migraine criteria for migraine-like headache attack in experimental studies

Diagnose	Migraine-like attack after pharmacological provocation
**Diagnostic criteria**	Headache fulfilling ICHD-II criteria C and D for migraine without aura
	**C)** Headache has at least two of the following characteristics Unilateral location Pulsating quality Moderate or severe pain intensity (>4 on VRS) Aggravation by cough (in-hospital phase) or causing the avoidance of routine activity (out-hospital phase) (e.g. walking or climbing stairs)
	**D)** During headache at least one of the following Nausea and/or vomiting Photophobia and phonophobia
	Headache described as mimicking usual migraine attack and treated with triptan/aspirin

Taken and adapted from Headache Classification Committee of the International Headache Society (2004) and Ashina *et al.* (2013). VRS = Verbal Rating Scale.

At the end of the study recording, rescue medication for pain and/or nausea with paracetamol (acetaminophen), ibuprofen, sumatriptan, aspirin, domperidone and ondansetron were additionally available.

#### Statistical analysis

Associations between qualitative variables were analysed with the Cohen’s kappa measure of association. Binary logistic regression was used to analyse the effect of headache frequency, age, years with migraine, and diagnosis on allodynia triggering, and presented as odds ratios (OR) and confidence intervals (CI). Analyses for the reproducibility of allodynia triggering were only performed for up to two exposures to nitroglycerine. The differences in allodynia response to the two different treatments were not statistically tested here due to small sample size in each subgroup. Significance was assessed at the *P* < 0.05 level (IBM-SPSS 24.0).

### Preclinical studies

All experiments were conducted in compliance of a research protocol approved by the UCSF or NYU Institutional Animal Care and Use Committee, and conforming to the National Institute of Health Guide for the Care and Use of Laboratory Animals, adhering to ARRIVE guidelines and those of the Committee for Research and Ethical Issues of IASP (Zimmermann, 1983).

#### Animals and experimental design

The animals per group was based on published data and previous experience that typically sees difference in means of 25–30% [standard deviation (SD) = 15–20%] with a two-sided alpha of 0.05 and power of 80%, which requires sample size of 10–12 animals to measure time points up to 3 h. We used male Sprague-Dawley rats (250–390 g) throughout these studies. Prior to the day of study they were housed in temperature and light controlled rooms for at least 7 days, with *ad libitum* access to food and water. Each experimental group within a series was assigned an integer and all rats were randomly assigned to the different experimental protocols and drug groups using the Microsoft Excel random number generator with the parameters set by the number of groups. We worked through these groups in the order assigned by the random number generator and studies were operator blinded with respect to chemical intervention, to avoid bias.

#### Animal preparation and electrophysiological recording

The surgical preparation, physiological monitoring, electrophysiological recording methods and analyses are the same as those reported in detail previously (Akerman *et al.*, 2010; Akerman and Goadsby, 2015). Briefly, rats were anaesthetized with sodium pentobarbital (Nembutal®, 60–70 mg/kg, intraperitoneal) and maintained with propofol (PropoFlo^TM^, 15–25 mg/kg/h i.v. infusion), and prepared for electrophysiological extracellular recording of dural-responsive neurons in the trigeminocervical complex. Blood pressure, body temperature and expired CO_2_ were continuously monitored and kept within physiological limits. During electrophysiological recording animals were paralysed with pancuronium bromide (Pavulon®, Organon) 0.4 mg initially and maintained with 0.2 mg every 35 min. A sufficient depth of anaesthesia was judged by the absence of paw withdrawal and corneal blink reflex, and during muscular paralysis by fluctuations of blood pressure and changes to expired CO_2_.

#### Characterization of neurons

A tungsten recording electrode (0.5–1 MΩ, tip diameter 0.5 µm) was advanced into the trigeminocervical complex region at 5-µm increments. Neuronal responses were characterized for their cutaneous and deep receptive field, and were assessed through all three trigeminal territories for both noxious and non-noxious responses, as the recording electrode advanced through the spinal cord. Neurons identified as having cutaneous receptive fields within the ophthalmic facial dermatome were then tested for convergent nociceptive input from the trigeminal innervation of the dura mater. Nociceptive-responsive trigeminal afferents were activated using electrical stimulation of the dura mater using square-wave stimuli (100–200 µs pulse, 0.25 Hz and 8–15 V). By moving the bipolar stimulating electrode around the dural receptive field and recording responses to electrical stimuli we were able to establish its optimal position to produce reliable and significant evoked action potential spikes. The distinction of trigeminal Aδ (‘fast’ responses) and C-fibres (‘slow’ responses) that innervate the dura mater is based on the approximate conduction velocities of Aδ (2.0–30.0 m/s) and C-fibres (0.5–2.0 m/s) (Millan, 1999) and the distance from the dural stimulation site to the trigeminocervical complex recording site (30–40 mm) (Akerman and Goadsby, 2015). Facial receptive field characterization consisted of 10 brush strokes applied to the facial receptive field over 7–8 s for the innocuous response, and pinch with forceps for 4 s for the noxious response. Spontaneous activity (spikes per second, Hz) was recorded throughout and measures for analysis taken for 300 s preceding the dural stimulation. Post and peri-stimulus time histograms of neural activity were displayed and analysed using Spike2 v8.

#### Drugs

Nitroglycerine (American Regent, Inc) was dissolved in 30% alcohol, 30% propylene glycol and water in a stock solution of 5 mg/ml. Nitroglycerine (10 mg/kg) was administered (0.6 ml, s.c.) at the neck scruff, given slowly over 2–3 min, and neural responses monitored for 3 h. This dose has been shown previously to be optimal to produce neuronal activation along the migraine pain pathway in rats (Tassorelli and Joseph, 1995) and craniofacial allodynia (Marone *et al.*, 2018). Nitroglycerine (1 mg/kg, s.c.), was used as a low dose control, and vasoactive intestinal peptide (VIP, Tocris Cookson Ltd) (Akerman and Goadsby, 2015), dissolved in saline (150 µg/kg, s.c.), was used as a control for the potential vascular effects of nitroglycerine. Naratriptan hydrochloride (Connor *et al.*, 1997; Cumberbatch *et al.*, 1998) (10 mg/kg in water; GlaxoSmithKline) was used to determine if a triptan can reverse the nitroglycerine-induced trigeminovascular changes, 135 min after nitroglycerine administration. We chose naratriptan as we have used this extensively in previous preclinical studies; doing so would thus allow us to compare our new data with those from previous studies. This, or similar doses, do not affect normal spinal nociceptive processing (Connor *et al.*, 1997; Cumberbatch *et al.*, 1998). This dose is similar to that used in previous preclinical studies that observed 50% or greater inhibition of neuronal responses that are prolonged for up to 45 min, and reflects the relative binding affinity at 5-HT_1B/1D_ rat receptors, which is lower compared to humans.

#### Statistical analysis

Analysis of blood pressure changes across groups was made using a one-way ANOVA followed by independent two-tailed *t*-tests, where necessary. The exact latency of neuronal discharges was established separately in each experiment and this latency window used throughout. The data collected from post-stimulus histograms represents the number of action potential spikes that fired within a latency window per stimulation, averaged over 20 stimulations (sweeps; spikes/sweep, s/s). Cutaneous receptive field responses and ongoing spontaneous neuronal activity are measured in cell firings per second (Hz). All data are expressed as mean ± standard error of the mean (SEM). Statistical analysis was performed using ANOVA for repeated measures with Bonferroni *post hoc* correction for multiple comparisons to measure the time course of significant drug intervention, using a 95% CI. If Mauchly’s test of sphericity was violated we made appropriate corrections to degrees of freedom according to Greenhouse-Geisser. Student’s paired two-tailed *t*-test for *post hoc* analysis was used to test for the time points of significance, using the average of the two or three baselines for comparison, again using the criteria of Bonferroni correction. Kruskal-Wallis test was used for non-parametric data, where applicable. Statistical significance was set at *P* < 0.05 (using IBM-SPSS 22.0 throughout).

### Data availability

The authors confirm that the data supporting the findings of this study are available within the article and its Supplementary material. Inquiries for additional data are available from the corresponding authors, upon reasonable request.

## Results

### Human studies and demographics

Of 335 patients prescreened for eligibility, 53 patients (18–50 years, mean = 35 years) with migraine with or without aura were recruited into the study, and each was exposed to nitroglycerine at least once, having provided informed consent. The range of previous headache days was 0–22 days per month (mean = 9 days). There were no contraindications to the use of nitroglycerine, aspirin or sumatriptan treatment. Twenty-three subjects reported cranial allodynia accompanying their spontaneous migraine (Table 2). Subject demographics are summarized in Supplementary Table 2.

**Table 2:** Summary of responses of all patients who reported spontaneous cranial allodynia with their migraine attacks

**Case #**	**Headache days per month (/30)**	**Triggered allodynia V1**	**Triggered allodynia V2**	**Triggered allodynia V**3	**Response to treatment**
1	10	N (HA)	-	-	-
2	22	Y	N	Y	Y (ASA)
5	10	Y	-	-	Y (ASA)
6	15	Y	Y	-	Y (ASA)
8	7	N (HA)	Y	Y	Y (ASA) Not V3
11	10	N (no HA)	-	-	-
12	8	Y (but no HA)	-	-	Not treated as no headache
17	10	N (HA)	-	-	-
21	3	N (HA)	-	-	-
24	10	Y	Y	Y	Y (ASA) Not V3
26	15	N (HA)	-	-	-
31	22	N (HA)	N (HA)	-	-
33	4	Y	Y	-	Y (Suma)
34	6	Y	Y	-	Y (Suma)
37	12	Y	-	-	Y (Suma)
42	6	Y	N (HA)	-	N (Suma)
43	16	Y	Y	-	Y (Suma) V1
45	15	N (HA)	-	-	-
46	2	Y	Y	-	N (Suma)
49	8	Y	-	-	Y (Suma)
50	12	Y	Y	-	Y (Suma) V1
51	5	N (no HA)	-	-	-
53	4	Y	Y	-	Y (Suma)

ASA = 1 g i.v. aspirin; HA = headache triggered with nitroglycerine; N = no; Suma = 6 mg sumatriptan s.c.; Y = yes with headache (HA) unless stated otherwise; V1–3 = visit number with nitroglycerine exposure.

Dash represents missing data for that individual. Sumatriptan treated patients only had two exposures to nitroglycerine.

### Nitroglycerine triggers cranial allodynia that responds to abortive treatment

#### Visit 1

Of the 53 subjects, 44 had migraine headache triggered with nitroglycerine and nine did not, although one had left cranial allodynia triggered without moderate-severe headache (Case 12); an 83% triggering rate. All cases (53/53; 100%) experienced a mild bilateral headache associated with the nitroglycerine infusion, which ceased soon after the infusion was stopped. In those who developed a migraine headache, this headache was delayed and worse in severity, as well as being associated with other migraine-defining symptomatology, as outlined in the modified criteria (Table 1). The time to onset of migraine headache when categorized as either moderate or severe intensity was 116.9 ± 10 min (range 20–278 min, *n* = 44). Overall, 17 subjects reported headache-associated cranial allodynia following nitroglycerine exposure (Tables 2 and 3). Subjects who reported cranial allodynia accompanying their spontaneous migraine attacks (13/20; 65%) were more likely to have allodynia triggered with nitroglycerine alongside their headache compared to those that did not report cranial allodynia (4/24; 16%), Cohen’s kappa measure of association, *k* = 0.445, *P* = 0.005, with 70% reliability. A significant number of these subjects (14/17 subjects, 82%, Cohen’s kappa measure of association, *k* = 0.815, *P* < 0.001) responded to treatment with either aspirin (*n* = 5/5) or sumatriptan (*n* = 9/12). Of note, the one subject who developed allodynia without headache following nitroglycerine exposure was not treated. There was no significant correlation between the reporting of spontaneous allodynia and the success in triggering headache with nitroglycerine (Cohen’s kappa = 0.155, *P* = 0.144); i.e. the rate of triggered headache reporting in the baseline allodynia and non-allodynic groups was similar.

**Table 3:** Summary of responses of all patients given nitroglycerine who did not previously report spontaneous allodynia

Case #	Headache days per months (/30)	Triggered allodynia V1	Triggered allodynia V2	Triggered allodynia V3	Response to treatment
3	2	N (HA)	N (HA)	N (HA)	-
4	1	N (HA)	N (HA)	-	-
7	15	Y	Y	Y	Y (ASA) Not V2
9	5	N (HA)	-	-	-
10	2	N (HA)	-	-	-
13	4	N (no HA)	-	-	-
14	12	N (HA)	N (HA)	N (HA)	-
15	7	N (no HA)	-	-	-
16	10	N (HA)	N (HA)	N (HA)	-
18	4	N (no HA)	-	-	-
19	6	N (no HA)	-	-	-
20	5	N (HA)	N (HA)	Y	Y (ASA)
22	12	N (HA)	-	-	-
23	6	N (HA)	-	-	-
25	0	N (no HA)	-	-	-
27	15	N (HA)	Y	N	N (ASA)
28	8	N (no HA)	-	-	-
29	4	N (HA)	-	-	-
30	16	N (HA)	N (HA)	-	-
32	15	Y	-	-	N (Suma)
35	4	N (HA)	Y	-	Y (Suma)
36	15	N (HA)	N (HA)	-	-
38	16	N (HA)	-	-	-
39	8	N (HA)	Y	-	Y (Suma)
40	10	Y	-	-	Y (Suma)
41	6	N (HA)	-	-	-
44	6	Y	Y	-	Y (Suma)
47	15	N (HA)	-	-	-
48	5	N (HA)	-	-	-
52	10	N (HA)	-	-	-

ASA = 1 g i.v. aspirin; HA = headache triggered with nitroglycerine; N = no; Suma = 6 mg sumatriptan s.c.; Y = yes with headache (HA) unless stated otherwise; V1–3 = visit number with nitroglycerine exposure.

Dash represents missing data for that individual. Sumatriptan treated patients only had two exposures to nitroglycerine.

#### Subsequent visits

On subsequent visits patients were blinded to either nitroglycerine or placebo. Based on study design, there was the opportunity for subjects to be exposed to nitroglycerine up to three times. Of the initial 44 subjects, 17 of whom had cranial allodynia, 24 were re-exposed to nitroglycerine a second time and nine a third time. Because of the sequentially reduced sample size across visits (due to drop outs and the mixed design regarding number of study visits), agreement analysis for allodynia reporting across visits was only performed for up to two nitroglycerine exposures. Of the 24 patients re-exposed to nitroglycerine, 23 developed migraine-like headache, and 14 of these had cranial allodynia triggered (*n* = 14/23, 61%). Within this group, 10/12 (83%) patients had allodynia triggered on their first exposure also had allodynia symptoms triggered on re-exposure, and 4/11 (36%) patients that did not have allodynia triggered on first exposure, did on the second (Cohen’s kappa = 0.5, *P* = 0.021, 72% reliability). Overall, 10 patients with allodynia symptoms responded to the abortive treatments (71%). Six patients had cranial allodynia triggered on their third visit (67%) and three responded to treatment (50%). Note that on all blinded trigger visits, no subjects developed migraine-like headache or cranial allodynia with placebo infusion.

#### Overall summary

Of 86 exposures to nitroglycerine across repeated visits, 76 were treated with either aspirin or sumatriptan, and 37 included subjects experiencing allodynia with migraine-like headache. Overall, in 28 of these 37 cases (76%) cutaneous allodynia was successfully treated, and in 15 of 21 cases (71%) sumatriptan successfully aborted allodynia. In all cases of allodynia but one, the allodynia was associated with moderate-severe headache either following or co-occurring with its onset. The occurrence of allodynia following nitroglycerine provocation in this study is therefore applicable to allodynia associated with spontaneous migraine attacks.

### Effects of diagnosis, age and headache days

A binary logistic regression model was fitted to the data to investigate the relationship between age, headache frequency, years with migraine and the outcomes of triggered allodynia and reporting spontaneous allodynia at Visit 1. There was no association of age and reporting of allodynia with spontaneous attacks (OR: 0.967, 95% CI: 0.880–1.062 *P* = 0.477), no association with headache frequency and reporting allodynia with spontaneous attacks (OR: 1.109, 95% CI: 0.970–1.268, *P* = 0.132). There was a significant association between reporting triggered allodynia and the presence of allodynia with spontaneous attacks (OR: 8.151, 95% CI: 1.984–33.479, *P* = 0.004). There was no association between years lived with migraine and spontaneous allodynia within this model (OR: 1.035, 95% CI: 0.944–1.136, *P* = 0.464). When the same analysis was done for the presence of triggered allodynia at Visit 1, there was no association of either age (OR: 1.051 95% CI: 0.958–1.154, *P* = 0.703) or headache days (OR: 1.028 95% CI: 0.892–1.184, *P* = 0.703). There was no effect of years lived with migraine on the reporting of triggered allodynia within this model (OR: 0.980, 95% CI: 0.896–1.073, *P* = 0.665). There was no effect of diagnosis, either episodic migraine without aura, episodic migraine with aura or chronic migraine, on reporting spontaneous triggered allodynia (*P* = 0.68). This fits with the regression analysis that there did not seem as strong an association of the positive reporting with triggered allodynia with increasing headache days.

### Preclinical studies

#### Neuronal identification and properties

**Figure 1:**
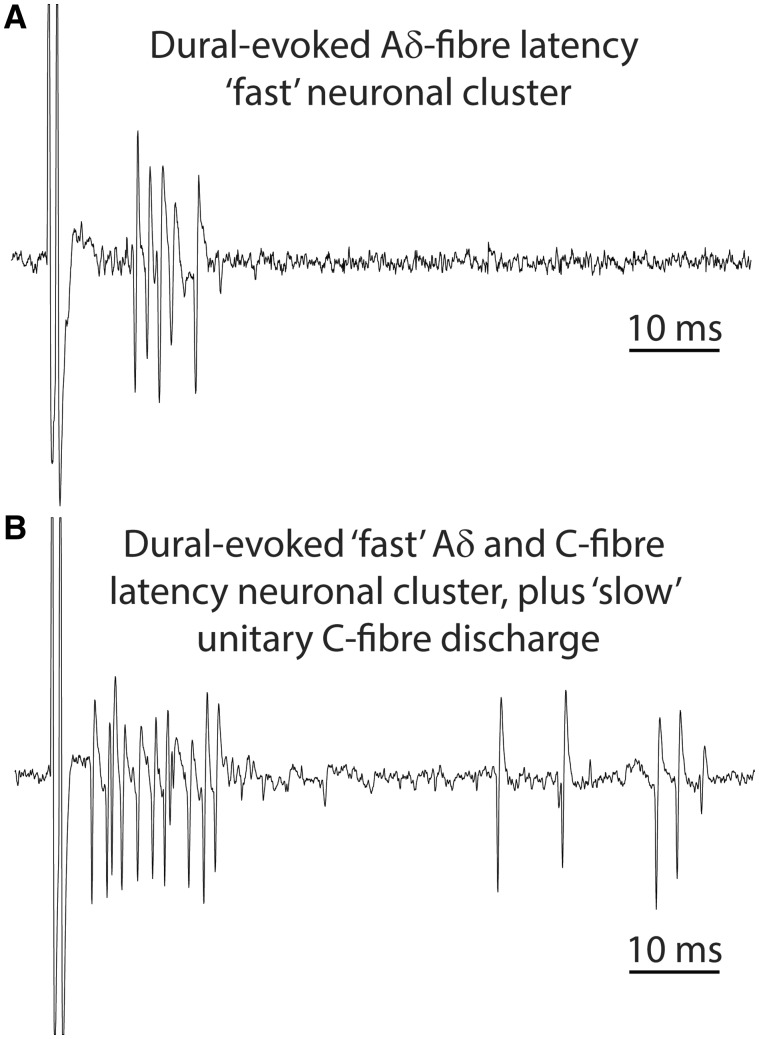
**Original tracings characterizing subtype of dural-evoked trigeminocervical neuronal fibres.** Example tracings illustrate (**A**) early reproducible neuronal discharges classified as receiving only Aδ-fibre input (<20 ms latency; ‘fast’ neuronal responses), and (**B**) reproducible neuronal spikes with extended discharges beyond 20 ms latency, which were classified as receiving both Aδ and C-fibre inputs (<30 ms latency; ‘fast’ neuronal responses), and also with later unitary discharges that were also classified as receiving C-fibre input (‘slow’ neuronal responses).

Electrophysiological recordings were made from 44 neurons that responded to electrical stimulation of the trigeminal afferent innervation of the dura mater, and with cutaneous facial receptor fields that included predominantly the first division of the trigeminal nerve, and on occasion also the second and third division. Of these 44, 29 (25 wide dynamic range, three high-threshold, and one low-threshold) exhibited a combination of either reproducible burst (action potentials in close proximity of each other) of discharges at 3–20 ms (Aδ fibre) or 3–30 ms (both Aδ and C-fibre), with also a unitary (single isolated action potentials) discharge at 20–80 ms (C-fibre), which were classified as receiving both Aδ and C-fibre inputs. Neuronal bursts (*n* = 15; all wide dynamic range) exhibiting only early discharge responses at 3–20 ms (Aδ fibre), were classified as receiving only Aδ-fibre inputs (Fig. 1A and B). Neurons were located in mainly nociceptive-specific superficial (laminae I and II) and deeper layers (laminae V and VI) of the dorsal horn of the trigeminocervical complex at range of depth of 200–1200 µm.

Through all animals/groups studied the average baseline ongoing spontaneous firing rate was 27.1 ± 2 Hz, and there was no significant difference across the different groups [*F*(3,40) = 2.7, *P* = 0.062]. There was also no significant difference at baseline between all groups for dural-evoked Aδ-fibre units [*F*(3,43) = 1.2, *P* = 0.3], or unitary C-fibre discharges [*F*(3,15) = 1.2, *P* = 0.35], with an average baseline of 7.9 ± 0.2 and 1.3 ± 0.2 action potential spikes per sweep (s/s), respectively. There was also no significant difference at baseline between groups in response to both innocuous (*H*_3_ = 4.3, *P* = 0.23) and noxious (*H*_3_ = 0.6, *P* = 0.91) somatosensory stimulation of the facial receptive field. The physiological characteristics of this neuronal population are similar to previous studies (Akerman and Goadsby, 2015).

#### Blood pressure responses

Nitroglycerine (1 and 10 mg/kg) and VIP (150 µg/kg) each caused a significant drop in blood pressure (range 8.7–53.9 mmHg) upon administration. There was no significant difference in the blood pressure drop between nitroglycerine (10 mg/kg) and both nitroglycerine [1 mg/kg, *t*(33) = 1.9, *P* = 0.071] and VIP [*t*(33) = 1.6, *P* = 0.13]. There was also no difference in the duration of the blood pressure drop before it returned to baseline across the three groups [*F*(2,43) = 0.23, *P* = 0.78], which was 1323.6 ± 91 s across all groups.

### High-dose nitroglycerine causes delayed activation of central trigeminocervical neurons

**Figure 2:**
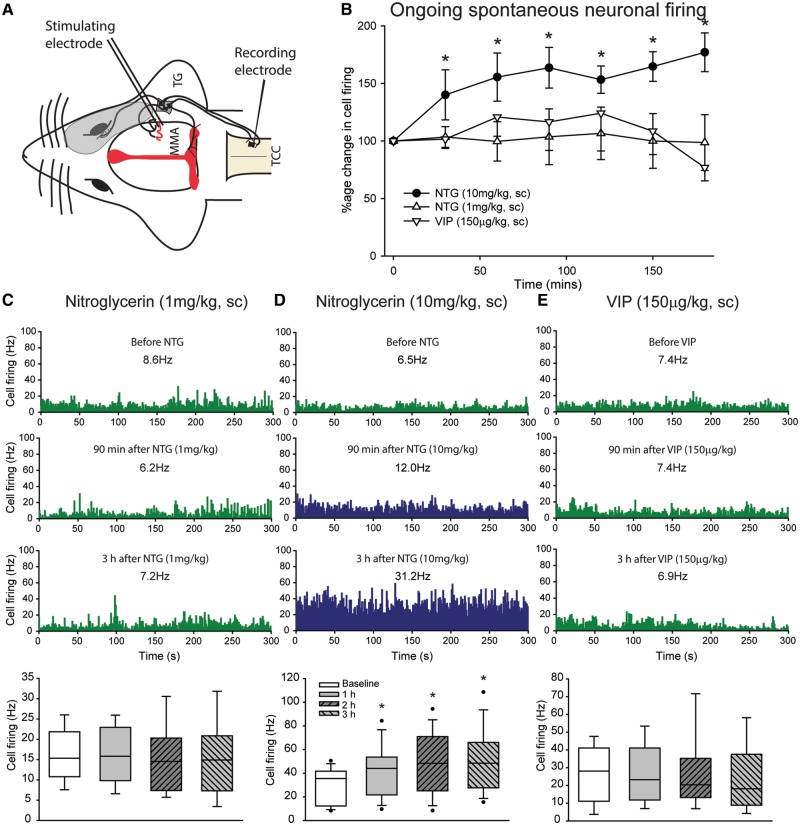
**High dose nitroglycerine causes delayed activation of central trigeminocervical neurons.** (**A**) Experimental set-up for electrophysiological recording of neurons in the trigeminocervical complex (TCC), which respond to electrical stimulation of the trigeminal innervation of the dural meninges, with a cutaneous facial receptive field (shaded area) characterization in the ophthalmic dermatome. (**B**) Time course changes in ongoing spontaneous trigeminocervical neuronal firing [action potentials per second (Hz)] in response to nitroglycerine (NTG, 1 and 10 mg/kg, s.c.) and vasoactive intestinal peptide (VIP, 150 µg/kg, s.c.). The data have been normalized to represent the percentage change from baseline, and are expressed as mean ± SEM. (**C**–**E**) Representative peri-stimulus time histograms with a single animal for each group demonstrating ongoing spontaneous trigeminocervical neuronal firing before (**C**) nitroglycerine (1 mg/kg), (**D**) nitroglycerine (10 mg/kg) and (**E**) VIP administration, and at 90 min and 3 h post-infusion. The numbers indicate the mean firing [spikes per second (Hz)] over the displayed time period, green neuronal firing indicates baseline and no change in responses, blue neuronal firing indicates a significant increase in neuronal firing. Beneath each of these is a box-and-whisker plot representing median and interquartile range (IQR), with 5th and 95th percentile, and individual points beyond this, of all trigeminocervical complex neurons studied per group, sampled at 1 h intervals after drug administration. Nitroglycerine (10 mg/kg, *n* = 14) caused a delayed and significant increase in ongoing spontaneous trigeminal neuronal firing that is significant (*P < 0.05) after 60 min and continues until at least 3 h, whereas nitroglycerine (1 mg/kg, *n* = 9) and VIP (*n* = 9) do not cause any changes over 3 h. MMA = middle meningeal artery; TG = trigeminal ganglion.

Overall nitroglycerine (10 mg/kg) caused a significant increase in ongoing spontaneous trigeminal neuronal firing over the first 60 min [*F*(3.1,40.9) = 3.7, *P* = 0.018, *n* = 14, 31.2 ± 4 Hz to 42.0 ± 6 Hz], specifically after 30 min [*t*(13) = 3.2, *P* = 0.007], which remained significant up to at least 3 h [*F*(6,78) = 7.3, *P* = 0.001, up to 50.4 ± 7 Hz; Fig. 2A–E]. Using the critical ratio test (Nagler *et al.*, 1973), which in effect implies that a 30% change from baseline is considered significant, and 10–30% change unclassified, in eight rats there was no change in ongoing firing after 30 min. These included six neuronal responses classified as receiving both Aδ and C-fibre inputs, and two with Aδ-fibre only, all wide dynamic range. At 1 h there were still four rats that had not reached this critical ratio. However, across the 3 h, all neuronal responses exhibited a significant increase from baseline based on this critical ratio. Neither nitroglycerine [1 mg/kg, *F*(1.9,15.4) = 0.28, *P* = 0.75, *n* = 9] nor VIP [150 µg/kg, *F*(1.8,14.3) = 0.92, *P* = 0.41, *n* = 9] caused any increase in ongoing firing, over the first 60 min or up to 3 h (Fig. 2A–E), and in each group no animals exhibited a significant increase in ongoing firing based on critical ratio. Therefore, only the higher dose of nitroglycerine causes significant changes to ongoing spontaneous firing, and this was not driven by cardiovascular or cerebrovascular changes, whereas activity with lower dose nitroglycerine and VIP does not change beyond normal.

### High dose nitroglycerine causes delayed hypersensitivity of central trigeminocervical neurons to cephalic somatosensory stimulation

**Figure 3:**
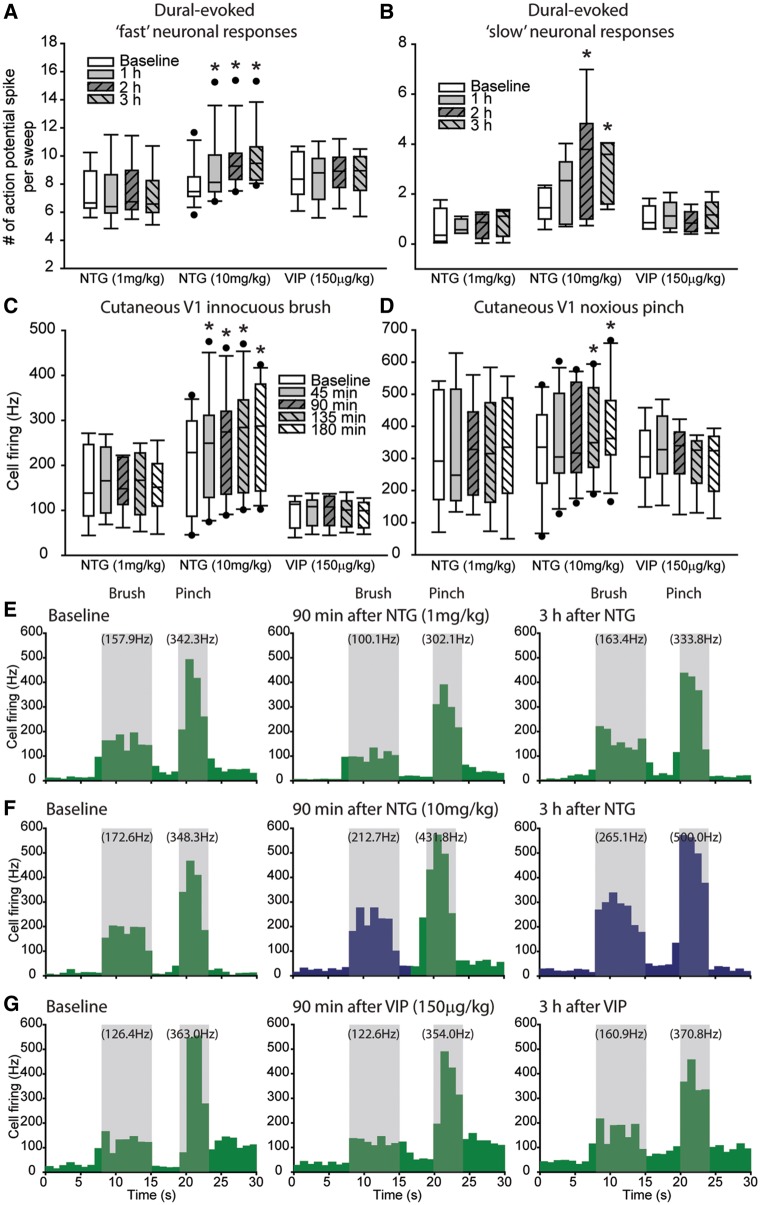
**High dose nitroglycerine causes delayed neuronal hypersensitive responses of central trigeminocervical neurons to somatosensory stimulation.** Box-and-whisker plots representing median and IQR, and the 5th and 95th percentile, with individual points beyond this, of (**A**) grouped data of intracranial dural-evoked bursts of trigeminocervical neuronal discharges that include inputs in the Aδ-fibre range (3–20 ms; ‘fast’ neuronal responses), and also those whose latencies were extended and therefore also receive inputs that include both Aδ and C-fibres (3–30 ms; ‘fast’ neuronal responses), and (**B**) intracranial dural-evoked unitary discharges within the C-fibre latency range (20–80 ms; ‘slow’ neuronal responses), in response to nitroglycerine (1 and 10 mg/kg, s.c.) and VIP (10 µg/kg, s.c.) administration. Nitroglycerine (10 mg/kg) caused a delayed hypersensitivity in neuronal responses to electrical stimulation of the trigeminal innervation of the dural meninges, for ‘fast’ (*n* = 14) and ‘slow’ (*n* = 7) neuronal responses that is significant compared to baseline after 1 and 2 h (*P < 0.05), respectively. Nitroglycerine (1 mg/kg) and VIP did not cause any change in neuronal responses. Box-and-whisker plots of (**C**) innocuous and (**D**) noxious cutaneous facial-evoked stimulation following nitroglycerine (1 and 10 mg/kg) and VIP. Representative peristimulus time histograms from a single animal for each group depicting trigeminocervical neuronal firing in response to innocuous brush and noxious pinch of the cutaneous facial receptor field before nitroglycerine (10 mg/kg, **E**), nitroglycerine (1 mg/kg, **F**) and VIP (**G**), and at 90 min and 3 h post-infusion. The numbers in parentheses indicate the mean firing (Hz) in response to stimulation (shaded area), green neuronal firing indicates baseline and no change in responses, blue neuronal firing indicates a significant increase in neuronal firing. Nitroglycerine (10 mg/kg) caused a delayed and significant increase in neuronal responses to innocuous and noxious facial cutaneous stimulation in the ophthalmic dermatome after 45 and 135 min, respectively. Nitroglycerine (1 mg/kg) and VIP did not cause any changes. *P < 0.05 significant compared to baseline.

This increase in ongoing neuronal firing manifested as significant hypersensitive responses of the same trigeminocervical neurons to dural stimulation. Nitroglycerine (10 mg/kg) caused a significant increase over 3 h in the number of dural-evoked action potential spikes for all neuronal discharges within the Aδ-fibre range, whose latency was extended to also include ‘fast’ C-fibre responses [*F*(2.7,36.7 = 14.7, *P* = 0.001, *n* = 14] that was significant after 60 min [*t*(13) = 3.6, *P* = 0.003, from 8.0 ± 0.4 to 9.2 ± 0.6 s/s], and remained significant until at least 3 h [*t*(13) = 8.0, *P* = 0.001, 9.8 ± 0.5 s/s]. There was also a significant increase in action potential spikes for unitary discharges within the ‘slow’ C-fibre range [*F*(6,36) = 3.5, *P* = 0.08, *n* = 7] that began after 90 min [*t*(6) = 2.4, *P* = 0.024, from 1.4 ± 0.3 to 3.5 ± 0.9 s/s] and remained significant until at least 3 h [*t*(6) = 2.7, *P* = 0.034, 2.6 ± 0.5 s/s]. Neither low dose nitroglycerine (1 mg/kg) nor VIP (150 µg/kg) had any significant effects on bursts of ‘fast’ Aδ/C-fibre neuronal discharges [nitroglycerine, *F*(6,48) = 0.7, *P* = 0.55, *n* = 9; VIP, *F*(6,48) = 0.7, *P* = 0.55, *n* = 9] or ‘slow’ C-fibre unitary discharges [nitroglycerine, *F*(2.0,5.9) = 0.6, *P* = 0.6, *n* = 4; VIP, not enough data for analysis] (Fig. 3A and B).

There was also a hypersensitive response to cutaneous facial stimulation after nitroglycerine (10 mg/kg), with increased neuronal firing to innocuous brush [*F*(1.9,18.8) = 24.4, *P* = 0.001, *n* = 11] and noxious pinch [*F*(4,40) = 7.0, *P* = 0.001, *n* = 11]. Neuronal responses to cutaneous innocuous brush [*t*(10) = 4.0, *P* = 0.001, *n* = 11] and noxious pinch [*t*(10 = 3.4, *P* = 0.007, *n* = 11] were significantly increased compared to baseline after 45 and 135 min, respectively, and continued until at least 3 h [Fig. 3C–G). However, nitroglycerine (1 mg/kg) and VIP did not cause hypersensitive neuronal responses to either innocuous brush [nitroglycerine, *F*(4,32) = 0.68, *P* = 0.61, *n* = 9; VIP, *F*(4,32) = 0.81, *P* = 0.53, *n* = 9] or noxious pinch [nitroglycerine, *F*(4,32) = 0.2, *P* = 0.94; VIP, *F*(1.7,13.2) = 1.8, *P* = 0.21] of the cutaneous facial receptive field.

### Naratriptan reverses nitroglycerine-evoked neuronal responses

**Figure 4:**
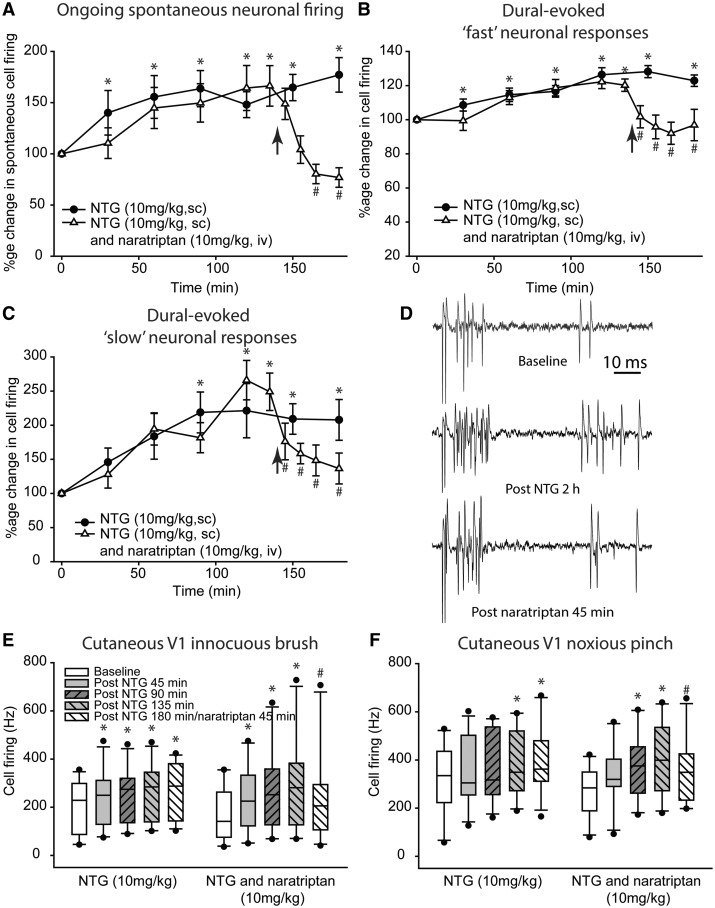
**Naratriptan reverses nitroglycerine-evoked activation of central trigeminovascular neurons and hypersensitive responses to somatosensory stimulation.** Time course of (**A**) the mean ongoing spontaneous trigeminal neuronal firing [action potential spikes per second (Hz)], (**B**) grouped data of intracranial dural-evoked bursts of trigeminocervical neuronal discharges that include inputs in the Aδ-fibre range (3–20 ms; ‘fast’ neuronal responses), and also those whose latencies were extended and therefore also receive inputs that include both Aδ and C-fibres (3–30 ms; ‘fast’ neuronal responses), and (**C**) intracranial dural-evoked unitary discharges within the C-fibre latency range (20–80 ms; ‘slow’ neuronal responses). All are in response to nitroglycerine (10 mg/kg, s.c.) and after treatment with the migraine abortive, 5-HT_1B/1D_ receptor agonist, naratriptan (10 mg/kg, i.v.). In each panel the data have been normalized to represent a percentage change from baseline and are expressed as mean ± SEM. Naratriptan significantly aborted the increase in ongoing spontaneous neuronal firing, and the hypersensitive neuronal responses to dural-intracranial electrical stimulation for ‘fast’ and ‘slow’ neuronal responses. (**D**) Original tracings of a single sweep (stimulus) in the same animal for a dural-evoked Aδ-fibre (‘fast’) neuronal burst and unitary C-fibre (‘slow’) discharge at baseline, 2 h after nitroglycerine administration, and 45 min after subsequent administration of the migraine abortive naratriptan. Both the Aδ-fibre neuronal burst and unitary C-fibre discharge become hypersensitive to dural stimulation after nitroglycerine, as evidenced by an increase in the number of action potential spikes at each latency. This is attenuated after treatment with naratriptan. Box-and-whisker plots representing median and IQR, and the 5th and 95th percentile, with individual points beyond this, of time course changes in trigeminocervical neuronal firing, in response to (**E**) innocuous and (**F**) noxious cutaneous stimulation of the facial receptive field after nitroglycerine, followed by treatment with naratriptan. The hypersensitivity in neuronal responses to both innocuous and noxious cutaneous facial stimulation 2 h after nitroglycerine is attenuated by naratriptan. *P < 0.05 represents a statistically significant difference compared to baseline (0 min). ^#^*P* < 0.05 represents a statistically significant difference compared to the time point just prior to administration of naratriptan, which was given post nitroglycerine 135 min. Arrowhead in **A**–**C** indicates time at which naratriptan was administered. All data are represented as mean ± SEM.

In a separate series of experiments we determined whether naratriptan (10 mg/kg, i.v.) is able to reverse the effects of nitroglycerine (10 mg/kg) on central trigeminovascular neurons, predicted to be effective based on clinical outcomes. Here, naratriptan was administered at the 135 min time point, and responses followed for a further 45 mins. Before conducting data analysis of the effects of naratriptan we first confirmed that nitroglycerine produced a statistically significant increase in neuronal response at 120 min (summarized in Table 4 and Fig. 4A–F). Subsequently, naratriptan significantly reversed the increase in ongoing spontaneous trigeminal neuronal firing [*F*(2.4,26.8) = 6.9, *P* = 0.002, *n* = 12], specifically after 25 min [*t*(11 = 3.0, *P* = 0.012] that was maintained through 45 min from 48.8 ± 9 Hz prior to naratriptan to 23.4 ± 5 Hz after 45 min. The dural-intracranial hypersensitive neuronal responses were also significantly inhibited by naratriptan for bursts of neuronal discharges within the Aδ-fibre range [3–20 ms; *F*(3.2,35.6) = 8.2, *P* = 0.001], specifically after 10 min [*t*(11 = 4.6, *P* = 0.001] that was maintained through 45 min (9.2 ± 0.6 prior to naratriptan to 7.4 ± 0.8 s/s after 45 min). Unitary discharges within the C-fibre range were also significantly inhibited by naratriptan [20–80 ms; *F*(2.3,11.7) = 4.2, *P* = 0.037, *n* = 6], specifically after 5 min [*t*(6 = 3.7, *P* = 0.014, *n* = 7] that was maintained through 45 min (4.5 ± 0.7 to 2.0 ± 0.4 s/s). The extracranial cutaneous facial hypersensitivity mediated by nitroglycerine was also significantly inhibited by treatment with naratriptan [*n* = 10, innocuous brush, *t*(9) = 3.8, *P* = 0.004; noxious pinch, *t*(9) = 2.9, *P* = 0.018].

**Table 4:** Summary of trigeminocervical neuronal data at baseline and after 2 h, in response to nitroglycerine (10 mg/kg, s.c.) for naratriptan group

**Naratriptan (10 mg/kg)**	
**Spontaneous activity (Hz)**	
Baseline	31.2 ± 4
Post nitroglycerine 2 h	47.5 ± 8 [*F*(2.2,24.6) = 5.8, *P = 0.007, *n* = 12]
**Dural-evoked Aδ and C-fibre clusters (AP/Stim)**	
Baseline	7.6 ± 0.4
Post nitroglycerine 2 h	9.3 ± 0.5 [*F*(5,55) = 9.6, *P = 0.001, *n* = 12]
**Dural-evoked unitary C-fibre discharges (AP/Stim)**	
Baseline	1.6 ± 0.3
Post nitroglycerine 2 h	4.0 ± 0.9 [*F*(5,25) = 8.1, *P = 0.001, *n* = 6]

AP/Stim = number of action potential spikes per stimulation; *P < 0.05 significance from baseline.

## Discussion

Here, we demonstrate reliable triggering of migraine headache with nitroglycerine and reproduction of cranial allodynia in patients with allodynia with their spontaneous migraine. Further, when patients were re-exposed to nitroglycerine on a second or third visit, there was >50% reproducibility of triggering cranial allodynia in these patients. The data with sumatriptan also clearly show allodynia can be aborted by a triptan in nitroglycerine-triggered migraine. Whilst the sample size of human subjects exposed to nitroglycerine here was not sufficient for extensive subgroup analysis of migraine subtypes, it is clear that nitroglycerine can acutely provoke cranial allodynia in humans and this is consistent with patient self-reports of acute allodynia associated with episodic migraine attacks and the lack of association of the presence of allodynia with effective treatment response (Cady *et al.*, 2009). In addition, these data complement similar data that triptans can abort cranial allodynia associated with spontaneous migraine (Goadsby *et al.*, 2008; Diaz-Insa *et al.*, 2011), and aspirin has a similar effect as shown in this study. Whilst the clinical study has been able to demonstrate a triptan and NSAID-response of nitroglycerine-induced cranial allodynia, apart from the response of allodynia to each treatment and the time to headache abortion, no additional differentiating data for the two treatment groups were obtained. Further, nitroglycerine can trigger activation and sensitization of trigeminocervical neurons in rat, which is not seen with the potent vasodilator VIP and this is also aborted with a triptan. The data suggest that nitroglycerine can be used as an effective and reliable trigger of migraine-associated cranial allodynia, and provides a valuable opportunity to study pathophysiological mechanisms related to cranial allodynia experimentally.

Triptan treatment has previously been shown to abort nitroglycerine-triggered migraine-like headache (Afridi *et al.*, 2005; Juhasz *et al.*, 2005). When administered either aspirin or sumatriptan in response to nitroglycerine-triggered migraine with cranial allodynia, the majority of the time patients responded to these treatments with abortion of both headache and cranial allodynia. No additional differentiating data for the two treatment groups were obtained. These findings are consistent with previous studies demonstrating that the NSAID ketorolac is able to terminate cutaneous facial allodynia (Jakubowski *et al.*, 2005*a*). However, several studies suggest that once cutaneous allodynia is established it responds much less to triptans (Burstein *et al.*, 2004; Jakubowski *et al.*, 2005*a*). It is hypothesized that this reflects the development of sensitization of central trigeminovascular neurons, and the consequent symptoms of extracranial cutaneous hypersensitivity (Burstein and Jakubowski, 2004; Levy *et al.*, 2004). It has led to a view in headache medicine that migraine responds most successfully when treated with triptans early into an attack, specifically before allodynia is established.

In a double-blind, placebo-controlled, parallel-group, multicentre trial spontaneous migraine treated with almotriptan early, when pain was mild, with the endpoint of pain-free by 2 h, was significantly more effective than when pain was moderate-severe. Furthermore, the presence of allodynia did not alter the efficacy of almotriptan to reach pain-free with early/mild or moderate/severe migraine. Allodynia was therefore not a reliable predictor of lack of response to a triptan in these spontaneous migraine attacks, but rather severity of pain (Goadsby *et al.*, 2008; Diaz-Insa *et al.*, 2011). These data are consistent with similar studies using rizatriptan (Cady *et al.*, 2007), and almotriptan and NSAIDs (Schoenen *et al.*, 2008). Moreover, severity of pain rather than time to treatment was the significant predictor of response across a meta-analysis of the eletriptan studies (Diener *et al.*, 2008). These data in spontaneous migraine share similarities with the nitroglycerine-triggered data we present, with triptan-responsive cranial allodynia accompanying migraine headache. The difference in response to triptans from these studies is a challenge. It may reflect variation both between patients and within patients from attack to attack.

Comparing data from different studies can be hazardous, and complicated by differing primary objectives and study design/methods. Focusing on pain severity, in the current study the criterion for treatment was when pain reached moderate-severe intensity, either with or without cranial allodynia, rather than early or late into an attack (117 ± 10 min). So, all triptan-responsive cranial allodynia was categorized as moderate-severe. In the triptan-unresponsive study, pain intensity was also likely categorized as moderate-severe throughout, whether treatment was early or late. However, in their early treatment group (1 h after pain onset) pain levels were statistically no different between the allodynic or non-allodynic groups. In the late treatment arm (4 h after pain onset) the allodynic group had significantly higher pain intensity (likely categorized as severe, 8.3 on a 0–10 scale) compared to the non-allodynic group (5.9) (Burstein and Jakubowski, 2004). It is not possible to determine whether this difference impacted the success of using a triptan, but in both treatment groups triptan use was significantly more likely to yield pain-free results in the non-allodynic group. It is also noteworthy that assessment of cranial allodynia was different through these studies. Burstein and colleagues (2004) used quantitative sensory testing (QST) measuring definitive cold, heat and mechanical pain thresholds. In our study, and the almotriptan study, cranial allodynia was assessed via a similarly phrased questionnaire. These surveys were both in line with validated questionnaires (Jakubowski *et al.*, 2005*b*; Ashkenazi *et al.*, 2007) determined to identify reliably allodynic or non-allodynic patients, compared to QST. We believe that our conclusion that nitroglycerine triggers triptan-responsive cranial allodynia in migraine patients is therefore valid compared to these studies.

Activation, or hypersensitive neuronal responses, of the dural trigeminovascular innervation is thought to be involved in the pathogenesis of throbbing migraine-like head pain (Penfield and McNaughton, 1940; Ray and Wolff, 1940). Likewise, symptoms of cranial allodynia in migraine are thought to be mediated by sensitization of these dural-responsive central trigeminovascular neurons that receive convergent inputs from facial cutaneous areas (Burstein *et al.*, 1998). These mechanisms can be modelled in animals using dural nociceptive activation, either via electrical stimuli (Goadsby and Zagami, 1991) or chemical mediators (Burstein *et al.*, 1998). A further aim of this study was therefore to determine that nitroglycerine mediates this underlying neurophysiological mechanism. We demonstrate that only high dose nitroglycerine causes a sustained increase in ongoing spontaneous firing of both Aδ and C-fibre trigeminal neurons that innervate the dural vasculature and cutaneous facial region. This manifests as prolonged neuronal hypersensitivity to cephalic intracranial-dural and extracranial-cutaneous somatosensory stimulation; a neuronal phenotype indicative of central trigeminovascular sensitization. To translate these data further, we found that naratriptan reversed intracranial-dural hypersensitivity. Naratriptan also reversed measures that reflect more intrinsic activity; increased ongoing spontaneous firing and hypersensitivity to cutaneous-facial stimulation. This suggests that triptans act via post-synaptic inhibition directly on second-order central neurons in the trigeminocervical complex, previously confirmed (Goadsby *et al.*, 2001), as well as peripherally to block pain signals from the dura mater to central trigeminovascular neurons. Overall, these data suggest that nitrergic-induced mechanisms can mediate the neurophysiological changes taking place along the migraine pain pathway, which result in throbbing head pain and cranial allodynia, and triptans can abort these mechanisms. This provides an explanation for the cephalic migrainous symptoms in nitroglycerine-mediated migraine, and potentially describes a mechanism in mediating spontaneous migraine with cranial allodynia that does respond to triptan.

Together, these data suggest that patients with migraine-associated cranial allodynia can be grouped into those responsive to triptan treatment, and those where cutaneous allodynia predicts lack of triptan efficacy. This points to at least two mechanisms in mediating the neurophysiological changes that result in migraine symptoms, and potentially distinguishing migraine subgroups. Further, preclinically there are now two approaches validated to help dissect and characterize the mechanism of these differences. First, placement of an inflammatory soup on the dura mater causes a neuroinflammatory cascade in the local dural microenvironment. This causes intrinsic phenotypic neuronal changes to central trigeminovascular neurons via activation and sensitization of primary trigeminal afferents, resulting in cephalic migraine symptoms (Strassman *et al.*, 1996; Burstein *et al.*, 1998). These intrinsic changes are unresponsive to triptan treatment (Burstein and Jakubowski, 2004). It seems that with nitroglycerine, and potentially many spontaneous migraine attacks, a different mechanism may be involved.

While we cannot definitively explain the mechanism of nitroglycerine-mediated migraine, several studies help us to exclude some options. Nitroglycerine is a potent vasodilator in humans (Hansen *et al.*, 2007; Schoonman *et al.*, 2008) and rodents (Akerman *et al.*, 2002), but previous studies confirm that dural vascular mechanisms are unlikely to mediate trigeminovascular sensitization (Akerman and Goadsby, 2015) and migraine symptoms (Amin *et al.*, 2013). Our data support this as neither low dose nitroglycerine nor VIP produced neuronal changes, despite similar vascular changes to high dose nitroglycerine. Importantly, VIP does not trigger migraine in migraine patients (Rahmann *et al.*, 2008). Nitroglycerine also mediates a dural inflammatory cascade, including upregulation of inducible NO synthase (iNOS), IL-1β, and IL-6 in dural macrophage, and dural plasma leakage (Reuter *et al.*, 2001). The nitroglycerine-induced plasma leakage was attenuated by a specific iNOS inhibitor, but iNOS inhibition was not efficacious in migraine treatment (Hoivik *et al.*, 2010; Hoffmann and Goadsby, 2012). Local inflammatory changes in the dural microenvironment are therefore probably not involved.

Nitroglycerine acts as a nitric oxide (NO) donor, which is a highly volatile agent that readily crosses the blood–brain barrier, diffusing into brain cells (Moncada *et al.*, 1991). It activates neuronal and pro-inflammatory mechanisms within various brainstem and diencephalic nuclei (Tassorelli and Joseph, 1995; Tassorelli *et al.*, 2007) involved in migraine pathophysiology, and thought to modulate trigeminovascular neurons. An increase in ongoing firing of neurons along the dural-trigeminal pain pathway is likely to translate to the development of intracranial headache, whereas intracranial hypersensitivity translates to the throbbing nature of pain and exacerbation of headache with physical activity. It is noteworthy that nitroglycerine seems to mediate differential responses on durally-innervated primary afferent and second-order trigeminovascular neurons. It does not alter ongoing firing of primary afferent neurons, despite causing intracranial hypersensitivity (Zhang *et al.*, 2013), whereas previous studies have shown, and we confirm, that it activates central trigeminovascular neurons (Koulchitsky *et al.*, 2004, 2009). The authors (Zhang *et al.*, 2013) speculate that nitroglycerine is primarily acting on central mechanisms, either second-order trigeminovascular neurons directly, or other central nuclei, to provoke the underlying neurophysiological mechanisms within trigeminovascular neurons, such as sensitization, which leads to migrainous symptoms, including enhanced response of peripheral noxious inputs. This may explain why the enhanced response of primary afferent neurons to nitroglycerine seems to be delayed beyond the time it mediates changes of central trigeminovascular neurons. These mechanisms are therefore more likely involved in mediating triptan-responsive cranial allodynia in spontaneous migraine.

There are important limitations of the translational data. The nitroglycerine doses in the preclinical study are considerably higher than those used in the clinical study. It is now acknowledged that human dosing does not directly translate to animal doses (Reagan-Shaw *et al.*, 2008). Many factors need to be taken into account to calculate the preclinical dose, including time to maximum effect, rate of metabolism, and body surface area. All usually increase the required effective dose in small animals. While the doses used are still relatively high, they are similar to previous studies (Tassorelli and Joseph, 1995; Marone *et al.*, 2018). Furthermore, our data are validated by the fact that the lower dose did not alter any measure of neuronal response, and the higher dose responded to triptan treatment, similar to that found in patients. Another issue relates to timing of response after nitroglycerine. Human studies generally report that migraine is delayed by 4–5 h after nitroglycerine. While we report the delay to moderate-severe headache of up to 278 min, the average time to reach moderate-severe head pain was actually 117 min, with the earliest time for a single patient at 20 min. Indeed, nearly one-quarter of the patients where migraine headache was triggered reached moderate-severe intensity by 60 min. In the preclinical study significant change in ongoing firing of central trigmeinovascular neurons was seen at 30 min, but in 8/14 rats this was not above a critical ratio (<30% increase). Indeed, even at 60 min there were still four animals where ongoing firing had not significantly increased. In a recent animal study reported, the authors used a human-like dose of nitroglycerine, and observed a delayed intracranial hypersensitivity in primary afferent neurons of 2–3 h (Zhang *et al.*, 2013), yet no observations of increase in ongoing discharge, a likely correlate of the development of intracranial headache, were observed. While the dose and the timings in our study do not directly overlap, we believe there is sufficient evidence to validate that the preclinical data translate to the findings clinically.

Another potential limitation to translating the data is that different triptans were used; the reasons for which are outlined in the ‘Materials and methods’ section. All triptans act via 5-HT_1B/1D_ receptors, with subtle variations in specific binding affinities, and efficacy at other 5-HT1 receptors (Tfelt-Hansen *et al.*, 2000). Naratriptan is described as having ‘a very similar pharmacological profile to that of sumatriptan’ (Connor *et al.*, 1997), but is perhaps a little more potent with better oral bioavailability. Naratriptan is also more lipophilic than sumatriptan, suggesting it may have greater brain penetrance, but definitive studies relating to this are not available. One of the major findings here is that sumatriptan inhibited nitroglycerine-induced cranial allodynia that accompanied migraine headache, and it has previously been shown to inhibit nitroglycerine-induced hind-paw allodynia in mice (Pradhan *et al.*, 2014). Allodynia is thought to be mediated by central neuronal sensitization. Therefore, despite potentially limited brain penetrance, sumatriptan is still able to abort these symptoms. Given that both sumatriptan and naratriptan were administered via parenteral routes, they have similar pharmacological profiles, and while naratriptan aborted signatures of trigeminovascular sensitization, sumatriptan aborted a likely phenotypic outcome, cranial allodynia, at the very least we believe that naratriptan is acting in a similar mechanistic way to sumatriptan to abort these neuronal and phenotypic outcomes. Therefore, we do not believe that using different triptans in the different arms of this study affected either the outcomes or their interpretation in this study.

In summary, it is clear that the migraineurs’ experience of their attack can be very individual, and this is reflected in how they respond to treatments. Nitroglycerine as a human model of migraine shares many phenotypic similarities common to spontaneous migraine, including now triptan-responsive cranial allodynia, which also translates into underlying mechanisms from preclinical studies. Combined with data from previous studies, it suggests there are several mechanisms mediating the underlying neurophysiological changes that result in migraine cephalic symptoms, translated by different preclinical approaches, where response to triptans is a differentiating feature. We clearly demonstrate a mechanism where migraine-associated cranial allodynia is successfully aborted by a triptan, which correlates with similar findings in spontaneous migraine. This contrasts with previous findings of a subgroup of patients where the presence of allodynia is predictive of triptans failing to provide adequate relief from migraine symptoms. We reflect that there is much to be gained in acknowledging that several preclinical approaches can, and should, be used to study pathophysiological mechanisms related to a disorder. Here we have validated an effective and reliable method to trigger experimentally migraine-like cranial allodynia that responds to abortive treatment, which shares a phenotype common to many spontaneous migraine attacks that is responsive to triptan treatment. This approach also translates preclinically and can therefore be used to dissect the neurophysiological mechanisms of sensitization relevant to provocative migraine studies, but also spontaneous migraine, and as a tool in drug development and discovery efforts.

## Supplementary Material

Supplementary Material

## Abbreviation

VIP: vasoactive intestinal peptide
