# Supplementary material for: Nitroglycerine triggers triptan-responsive cranial allodynia and trigeminal neuronal hypersensitivity
Source: Brain. 2018 Dec 27;142(1):103–19. doi: 10.1093/brain/awy313 (PMC6308314; doi:10.1093/brain/awy313)
Supplement: Supplementary Material [file awy313_supplementary_material.pdf]

Supplementary material for

Nitroglycerin triggers triptan-responsive cranial allodynia and trigeminal neuronal hypersensitivity

Authors: Simon Akerman[1,2,3*†], Nazia Karsan[4,5†], Pyari Bose[4,5], Jan R. Hoffmann[1,6], Philip R. Holland[4], Marcela Romero-Reyes[2,3] and Peter J. Goadsby[1,4,5*]

Corresponding authors. Dr. Simon Akerman (preclinical studies)

Email: sakerman@umaryland.edu

Professor Peter Goadsby (clinical studies)

Email: peter.goadsby@kcl.ac.uk

Figure S1: Timeline of visit 1, which reflects both study arms

Table S1: Full Inclusion/Exclusion Criteria

Table S2: Summary of the demographics of patients recruited into the study

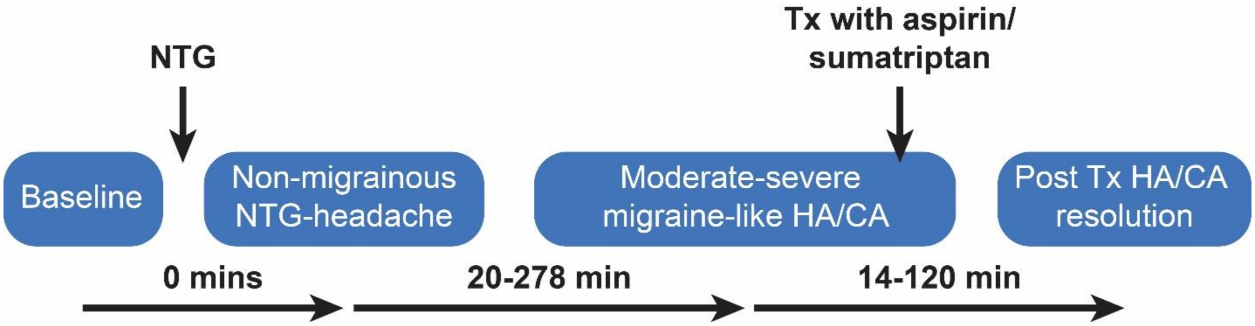

Figure S1. Timeline of visit 1

Each subject was exposed to a nitroglycerin (NTG) infusion where they likely experience immediate, non-migrainous headache, and subsequently may develop migraine-like headache (HA) symptoms and cranial allodynia (CA). If/when headache develops to moderate-severe intensity the patient was appropriately treated with either aspirin (1 g, IV) or sumatriptan (6 mg, SC). The symptoms are phenotyped as they develop throughout the study visit until post treatment (Tx) and recovery or up to at least 5 hours after the NTG infusion.

Table S1: Inclusion/Exclusion Criteria

Inclusion criteria

1. Subjects must be willing to travel to and from King's College Hospital during the course of a day
2. Diagnosis of migraine with or without aura, by the ICHD-3 beta criteria
3. Aged 18-50 years
4. Evidence of a personally signed and dated informed consent document indicating that the subject (or a legally acceptable representative) has been informed of all pertinent aspects of the trial
5. Willing and able to comply with scheduled visits, treatment plan, laboratory tests, and other trial procedures
6. Total body weight 45-100kg

Exclusion criteria

Subjects presenting with any of the following will not be included in the study

1. Allergy to aspirin, NTG or Sumatriptan
2. Concurrent anticoagulation with warfarin or novel anticoagulant
3. Concurrent treatment with methotrexate
4. History of past or current upper GI haemorrhage or GI ulceration
5. History of thrombocytopenia or haemorrhagic diathesis
6. Subjects with significant vomiting as part of their migraine attacks
7. ICHD3 beta criteria diagnosed medication overuse headache
8. Continuous or daily headache

9. Pregnancy and breastfeeding
10. Active menstruation at the time of the study
11. Aortic stenosis or significant hypotension (SBP<90mmHg or <100mmHg and symptomatic) precluding intravenous NTG infusion
12. Obstructive airways disease with previous exacerbation post aspirin, cardiac failure, renal failure or liver failure
13. Use of NSAID, aspirin or paracetamol analgesia within 24 hours of each visit
14. Regular treatment with Tramadol, codeine or other opioid drugs
15. Subjects who smoke more than 5 cigarettes a day or drink more than 6 cups of caffeinated drinks a day
16. History of psychosis or psychological disease either (a) requiring ongoing psychoactive drugs, or (b) that the Investigator has reason to believe will either affect the patient's neural pathways or hinder the performance of the patient with regard to perception of pain or ability to successfully complete the tasks required of them according to the protocol
17. Any person unable to lie still within the environment of the fMRI scanner for the required period to perform the study and those where MRI scanning is contraindicated (metal implants, pacemaker, etc.)
18. Any person unable to understand and follow the instructions of the investigators.
19. Any evidence of a history or current use of drugs of abuse.
20. Any subject unwilling or unable to conform to lifestyle guidelines
21. Known history of any other disorder which is strongly associated with polyneuropathy, including alcohol
22. Shift workers with altered circadian rhythm

23. Any other condition that at the opinion of the Investigator would make the subject unsuitable for the study

Lifestyle guidelines

1. Subjects will abstain from alcohol for 24 hours prior to each visit.
2. Subjects will abstain from caffeine-containing products for 12 hours prior to each visit.
3. Subjects will abstain from taking NSAIDs or paracetamol for 12 hours prior to each visit.
4. Subjects will abstain from the use of tobacco- or nicotine-containing products for 4 hours prior to admission until discharge for each visit.
5. Subjects should not smoke more than 5 cigarettes per day or consume more than 6 cups of caffeinated drinks per day for the duration of the study.
6. Subjects will be asked to maintain a normal sleep routine for the duration of the study.

Table S2: Summary of the demographics of patients recruited into the study

Case	Sex	Age	Diagnosis	Aura type	Headache days/month	# of study visits completed	Usual abortive medication	Usual preventive medication
1	F	50	EMO		10	1	Paracetamol/Naproxen	
2	F	47	CMA	Visual	22	4	Frovatriptan/Ibuprofen/ Paracetamol	Amitriptyline
3	M	28	EMA	Visual	2	4	Paracetamol/Aspirin	
4	M	43	EMA	Visual	1	2	Ibuprofen/Paracetamol	
5	F	48	EMO		10	1	Sumatriptan	Propranolol
6	F	41	EMO		15	2	Aspirin	
7	F	41	EMA	Visual	15	4	Naproxen/Codeine/ Paracetamol	
8	F	30	EMO		7	4	Paracetamol/Ibuprofen	
9	M	37	EMO		5	1	Ibuprofen	
10	F	40	EMO		2	1	Rizatriptan/Sumatriptan	
11	F	36	EMA	Motor	10	2	Paracetamol	
12	F	26	EMO		8	1	Paracetamol/Codeine/ Buclizine	
13	M	49	EMA	Visual/ speech	4	1	Ibuprofen	
14	F	45	EMA	Visual	12	3	Ibuprofen/Co-codamol/Naproxen	
15	F	20	EMA	Visual	7	1	Paracetamol/Codeine/ Buclizine	
16	F	27	EMA	Visual	10	4	Ibuprofen/Paracetamol	
17	F	27	EMA	Visual	10	1	Ibuprofen	
18	M	48	EMO		4	1	Ibuprofen	
19	F	35	EMO		6	1	Paracetamol/Codeine	
20	F	27	EMA	Visual	5	4	Paracetamol/Codeine/ Ibuprofen	
21	F	24	EMA	Visual	3	1	Ibuprofen	

22	M	46	EMO		12	1	Aspirin/Nurofen	
23	F	22	EMA	Visual	6	2	Paracetamol, Mefenamic acid	
24	F	37	EMO		10	4	Paracetamol, Ibuprofen	Pizotifen
25	F	36	EMA	Visual/ motor	0	1	Paracetamol	
26	F	29	EMA	Visual/ sensory	15	2	Sumatriptan/Ibuprofen	Propranolol
27	F	30	EMA	Visual/ sensory /speech	15	2	Sumatriptan/Paracetamol /Ibuprofen/Codeine	Candesartan
28	F	27	EMO		8	1	Ibuprofen/Aspirin/ Paracetamol/ Caffeine/Sumatriptan	
29	F	36	EMA	Visual	4	1	Sumatriptan	
30	F	50	CMA	Visual	16	3	Paracetamol/Codeine/ Buclizine/Sumatriptan	
31	F	49	CMA	Visual/ motor/ sensory	22	3	Almotriptan/Paracetamol /Ibuprofen	
32	F	37	EMA	Visual	15	2	Sumatriptan/Paracetamol /Nurofen	
33	F	36	EMA	Visual	4	3	Rizatriptan	Candesartan
34	F	25	EMA	Visual	6	3	Sumatriptan/Paracetamol /Ibuprofen	Amitriptyline
35	F	36	EMA	Visual/ sensory	4	3	Sumatriptan	Pizotifen
36	M	45	EMO		15	3	Zolmitriptan	Propranolol
37	F	45	EMA	Visual	12	3	Rizatriptan/Naproxen/ Paracetamol	Topiramate
38	F	35	CMA	Visual	16	2	Anadin/Sumatriptan	Topiramate
39	F	27	EMO		8	3	Sumatriptan	Propranolol
40	F	36	EMA	Visual	10	1	Paracetamol/Codeine/	

							Buclizine	
41	F	34	EMO		6	1	Paracetamol/Codeine/ Buclizine	
42	F	44	EMO		6	3	Rizatriptan/Naproxen	
43	F	18	CM		16	3	Sumatriptan	
44	M	43	EMO		6	3	Sumatriptan	
45	F	40	EMA	Visual	15	1	Rizatriptan/Paracetamol	Topiramate
46	F	35	EMA	Visual	2	3	Paracetamol/Ibuprofen	
47	F	21	EMO		15	3	Rizatriptan/Paracetamol/ Codeine	
48	F	24	EMA	Visual/ sensory /speech	5	2	Sumatriptan/Aspirin/ Codeine	Amitriptyline
49	M	42	EMO		8	1	Sumatriptan/Ibuprofen/ Paracetamol	
50	F	48	EMA	Visual	12	1	Sumatriptan/Naproxen	
51	F	26	EMA	Visual/ sensory	5	1	Diclofenac	Atenolol
52	F	27	EMA	Visual	10	1	Sumatriptan/Aspirin/Para cetamol/Ibuprofen	
53	F	30	EMO		4	1	Sumatriptan	

EMA, episodic migraine with aura; EMO, episodic migraine without aura; CMA, Chronic migraine with aura; CMO, Chronic migraine without aura
